# Serglycin Is Involved in TGF-β Induced Epithelial-Mesenchymal Transition and Is Highly Expressed by Immune Cells in Breast Cancer Tissue

**DOI:** 10.3389/fonc.2022.868868

**Published:** 2022-04-14

**Authors:** Marta Tellez-Gabriel, Xavier Tekpli, Trine M. Reine, Beate Hegge, Stephanie R. Nielsen, Meng Chen, Line Moi, Lisa Svartdal Normann, Lill-Tove R. Busund, George A. Calin, Gunhild M. Mælandsmo, Maria Perander, Achilleas D. Theocharis, Svein O. Kolset, Erik Knutsen

**Affiliations:** ^1^ Department of Medical Biology, Faculty of Health Sciences, UiT-The Arctic University of Norway, Tromsø, Norway; ^2^ Department of Medical Genetics, Oslo University Hospital, Oslo, Norway; ^3^ Department of Interphase Genetics, Institute for Cancer Genetics and Informatics, Oslo University Hospital, Oslo, Norway; ^4^ Department of Translational Molecular Pathology, The University of Texas MD Anderson Cancer Center, Houston, TX, United States; ^5^ Department of Clinical Pathology, University Hospital of North Norway, Tromsø, Norway; ^6^ Department of Tumor Biology, Institute for Cancer Research, Oslo University Hospital, Oslo, Norway; ^7^ Department of Research and Innovation, Vestre Viken Hospital Trust, Drammen, Norway; ^8^ Center for RNA Interference and Non-Coding RNAs, The University of Texas MD Anderson Cancer Center, Houston, TX, United States; ^9^ Biochemistry, Biochemical Analysis & Matrix Pathobiology Research Group, Laboratory of Biochemistry, Department of Chemistry, University of Patras, Patras, Greece; ^10^ Department of Nutrition, University of Oslo, Oslo, Norway; ^11^ Centre for Clinical Research and Education, University Hospital of North Norway, Tromsø, Norway

**Keywords:** proteoglycans (PG), serglycin (SRGN), transforming growth factor beta (TGF- β), epithelial-mesenchymal transition (EMT), breast cancer, tumor infiltrating immune cells, Single cell sequencing

## Abstract

Serglycin is a proteoglycan highly expressed by immune cells, in which its functions are linked to storage, secretion, transport, and protection of chemokines, proteases, histamine, growth factors, and other bioactive molecules. In recent years, it has been demonstrated that serglycin is also expressed by several other cell types, such as endothelial cells, muscle cells, and multiple types of cancer cells. Here, we show that serglycin expression is upregulated in transforming growth factor beta (TGF-β) induced epithelial-mesenchymal transition (EMT). Functional studies provide evidence that serglycin plays an important role in the regulation of the transition between the epithelial and mesenchymal phenotypes, and it is a significant EMT marker gene. We further find that serglycin is more expressed by breast cancer cell lines with a mesenchymal phenotype as well as the basal-like subtype of breast cancers. By examining immune staining and single cell sequencing data of breast cancer tissue, we show that serglycin is highly expressed by infiltrating immune cells in breast tumor tissue.

## Introduction

The extracellular matrix (ECM) is important in regulating cellular behavior and in diverse processes such as development, tissue formation, inflammation, and disease. The ECM is a complex tissue constituent, where its make-up varies between tissues and cells A major component of the ECM is the collagens, comprising more than 20 different members with large structural and functional differences. Other important ECM members are fibronectin, laminin, elastin, and proteoglycans (PGs) (1). The latter family of macromolecules contains both extracellular types such as agrin, versican, perlecan, and decorin, whereas important cell surface PGs are syndecans and glypicans. Serglycin is an intracellular PG, but with important extracellular functions when released from e.g. immune cells (2). PGs are unique among the ECM family of macromolecules as they are heavily glycosylated and sulfated, which contribute to their unique structures and functions. The core proteins are decorated with negatively charged glycosaminoglycan (GAG) chains, which are important for the interactions with partner molecules such as growth factors, proteases, and chemokines (3).

The role of PGs has been studied in relation to cartilage and bone functions, regulation of blood coagulation, extravasation of immune cells, cardiovascular disease, and cancer (4). Serglycin was initially defined as a hematopoietic PG (5), and a series of studies have focused on the role of serglycin in inflammation (2). However, serglycin has now been demonstrated to be expressed by diverse cell types such as muscle cells, chondrocytes, endothelial cells, and several types of cancer cells (6). Several PGs have been associated with epithelial-mesenchymal transition (EMT) (7–23), a naturally occurring, transdifferentiation program by which polarized epithelial cells lose their adherent and tight cell–cell junctions, enhance their migratory capacity, and increase their resistance to apoptosis (24). EMT is important in embryonic development, wound healing, and mammary gland development, and it is induced by a range of extracellular cues including a variety of growth factors, like transforming growth factor beta (TGF-β), and malignancy-associated cellular stress (25–27). The transition is orchestrated by several transcription factors, including Snail, Slug, TWIST, and ZEB (26). In cancer, the process of EMT is of fundamental importance, as EMT has been associated with dissemination, invasion, metastasis to distant organs, chemotherapy resistance, dormancy, and relapse (28–30). Overexpression of serglycin in the breast cancer cell line MCF7 induced expression of mesenchymal markers fibronectin and vimentin and the EMT transcription factor Snail, accompanied with a change to a more mesenchymal like morphology (31). Further, drug resistance towards the chemotherapeutics doxorubicin and methotrexate was increased with induced expression of serglycin (31). Using immunohistochemistry, serglycin was demonstrated to be present in breast cancer tissue, and serglycin was highly expressed in the aggressive MDA-MB-231 cell line (32). The MDA-MB-231 breast cancer cell line has been associated with a mesenchymal phenotype and has a high migratory potential (33, 34). High serglycin expression was also observed in highly metastatic nasopharyngeal cells (35). Furthermore, invasiveness and mobility of the cells was shown to be dependent on the glycosylated form of serglycin in breast cancer (32). In addition, the implication of serglycin in highly aggressive tumor cells was also studied in the glioblastoma cell line LN-18 (12). Silencing of the serglycin gene (*SRGN*) resulted in less proliferation, colony formation, stemness accompanied by astrocytic differentiation, and diminished tumor cell growth *in vivo*.

In this study, we have examined several TGF-β induced EMT RNA-Seq experiments, and we find that serglycin is upregulated by TGB-β stimulation. Disruption of the open reading frame (ORF) of the serglycin gene, *SRGN*, resulted in reduced and delayed induction of EMT. In breast cancer cell lines, serglycin expression is significantly associated with a mesenchymal phenotype. However, *in vivo*, we find serglycin expression to be highly related to infiltration of immune cells in the breast tumor tissue. Here, we have examined mRNA expression of serglycin in two breast cancer cohorts and a single cell sequencing data set, as well as the protein expression in a tumor microarray (TMA) by immunohistochemistry. Overall, our results presented show that serglycin is one of many important molecules in the complicated network of factors determining the transition from the epithelial to the mesenchymal phenotype, and suggest that serglycin in tumor tissue is mainly produced by infiltrating immune cells.

## Material And Methods

### Cell Culturing

BT474, BT549, Hs578T, MCF7, MDA-MB-231, MDA-MB-468, SK-BR-3, and T-47D, cells were purchased from the American Type Culture Collection (ATCC). HMLE cells were a kind gift from Robert Weinberg, Whitehead Institute for Biomedical Research and Department of Biology, Massachusetts Institute of Technology. BT474, BT549, MDA-MB-231, MDA-MB-468, SK-BR-3, and T-47D were cultured in RPMI 1640 (Sigma-Aldrich) supplemented with 10% fetal bovine serum (FBS) (Biochrom) and 1% penicillin-streptomycin (Sigma-Aldrich). BT549 cells were grown in the presence of 0.001 mg/ml insulin (Sigma-Aldrich) and T-47D were grown in the presence of 0.006 mg/ml insulin (Sigma-Aldrich). Hs578T were cultured in Dulbecco’s Modified Eagle’s Medium (DMEM; Sigma-Aldrich) supplemented with 10% FBS, 1% penicillin-streptomycin, and 0.01 mg/ml insulin. MCF7 were cultivated in Minimum Essential Medium Eagle (MEM; Sigma-Aldrich) supplemented with 10% FBS, 1% penicillin-streptomycin, and 0.01 mg/ml insulin. HMLE cells were grown in a 1:1 mixture of MEBM (Lonza) with DMEM/F12 (Sigma-Aldrich) supplemented with 10 ng/ml EGF, 0.5 μg/ml hydrocortisone, 0.01 mg/ml insulin, and 1% penicillin-streptomycin. All cell lines were incubated in a 5% CO2 humidified incubator at 37°C.

### Generation of Epithelial and Mesenchymal Cell Subpopulations of HMLE Cells

HMLE cells were separated into an epithelial and a mesenchymal subpopulation by immunomagnetic separation. Magnetic beads (Immunomagnetic M450 Dynabeads^®^, ThermoFisher Scientific) were coated with anti-EpCAM (MOC31, IQ Products). Trypsinized cells (1 ml) were mixed with 30 μl of coated beads and incubated on a rotating rack at 4°C for 30 min. The beads with epithelial cells and cell suspension containing mesenchymal cells were subsequently separated using a magnet rack.

### RNA Isolation, cDNA Synthesis, and RT-qPCR

From the breast cancer cell lines, RNA was isolated using the GenElute™ Mammalian Total RNA Miniprep Kit (Merck) according to the protocol provided by the manufacturer.

cDNA synthesis of total RNA was performed with SuperScript™ VI Reverse Transcriptase (ThermoFisher Scientific). 5.0 μM of random hexamer primer (P/N100026484, ThermoFisher Scientific) and approximately 200 ng of template were used for the reaction.

For RT-qPCR of cDNA from total RNA, 5 ng cDNA was mixed with FastStart Essential DNA Green Master (Roche Life Science) and 0.25 μM forward and reverse primer. All primer sequences are provided in **Table 1**. The LightCycler^®^ 96 was used for quantification, and the formulas 2^-ΔΔCq^ or 2^-ΔCq^ were used to calculate fold change or expression values, respectively, using GAPDH as an internal reference.

**Table 1:** Primer list used for RT-qPCR.

Gene	Forward primer sequence	Reverse primer sequence
***SRGN***	GCTACTCAAATGCAGTCGGC	CCCATTGGTACCTGGCTCTC
***CDH2***	GGCTTCTGGTGAAATCGCAT	AAGAGGCTGTCCTTCATGCAC
***VIM***	GAACGCCAGATGCGTGAAAT	AAGGTGACGAGCCATTTCCT
***TWIST***	ATTCAAAGAAACAGGGCGTGGG	AGAATGCAGAGGTGTGAGGATG
***ZEB1***	GCGCTTCTCACACTCTGG	GCGCTTCTCACACTCTGG
***ZEB2***	ACTTGCAGAGCATTACCCC	ACTTGCAGAGCATTACCCC
***GAPDH***	GAGCGAGATCCCTCCAAAAT	AAATGAGCCCCAGCCTTCT

### Transient Transfections

Lipofectamine^®^ 2000 (ThermoFisher Scientific) was used for transfection according to the protocol provided by the manufacturer. 25 μM siRNA or control siRNA was used for transfections. Cells were incubated for 48, 72, and 96 hours after transfection before harvesting. The following siRNA and control siRNA were used: siSRGN#1 (ThermoFisher Scientific, 4392420-S11041), siSRGN#2 (ThermoFisher Scientific 4392420-S11043), and Silencer^®^ Negative Control No. 1 siRNA, (ThermoFisher Scientific, AM4611).

### CRISPR/Cas9 Knockout

For generation of serglycin knockout cells, a synthetic single guide RNAs (sgRNAs) in complex with Cas9, targeting the protein coding sequence of *SRGN* (guide sequence: CTG AGT CTT ACC TTG AAC TGA GG, exon 1) were transfected by electroporation according to previously described protocol (36). sgRNAs and Cas9 2NLS Nuclease were purchased from Synthego and electroporation was performed using the Cell Line Nucleofector™ Kit V (Lonza). 100.000 cells were diluted in 50 µl electroporation buffer containing 3.6 µM of sgRNA and 0.8 µM Cas9 enzyme and electroporated using the program P-020 (Amaxa™ Nucleofector™ II). Cells were transferred immediately after electroporation to full media and left to recover for 1 week, before single cell colonies were generated by seeding of single cells using Flow cytometry. Screening for knockout clones were done by sanger sequencing of the target region using the forward primer AAA TGC AGT CGG CTT GTC CT and reverse primer CCC AAC AGT CAA AGG TGC CA. For sanger sequencing, the BigDye™ Terminator v3.1 Cycle Sequencing Kit (ThermoFisher Scientific) was used according to the manual provided by the manufacturer. Analysis of sanger sequences was done using the ICE Analysis by Synthego (https://ice.synthego.com/#/).

### TGF-β and EGF Stimulation

70.000 cells were seeded in a twelve well plate for each time point. Three wells per time point were stimulated with either 10 ng/ml recombinant human TGF-β (ThermoFisher Scientific, PHG9214) or 20 ng/ml EGF (R&B Systems). If reaching confluency, cells were split, and 70.000 cells were reseeded for each condition. Splitting was never performed less than three days before harvesting, to allow cells to recover.

### Analysis of Publicly Available RNA-Seq Data

Three publicly available RNA-Seq experiments where EMT had been induced by TGF-β were downloaded from the Sequence Read Archive (SRA) at NCBI. The bioproject numbers are: PRJNA474381 (37), PRJNA513977 (38), PRJNA260526 (39).

RNA-Seq analyses were performed with the CLC Genomic Workbench 21 following the RNA-Seq analysis pipeline. In short, raw reads were trimmed for quality and the presence of the Illumina adaptor, before being mapped to the human reference genome GRCh38 with Ensembl v104 annotation. Expression values were normalized as Trimmed Mean of the M-values (TMM) adjusted counts per million (CPM). Calculation of significantly differentially expressed genes was done using the Differential Expression for RNA-Seq tool provided in the CLC Genomic Workbench and were defined as >2.0 fold change with <0.05 FDR adjusted p-value.

### Gene Expression, Survival, Correlation, and Copy Number Analyses in Breast Cancer Cohorts

Breast cancer cell line RNA-Seq expression data and subtype information was downloaded from the Cancer Cell Line Encyclopedia (https://sites.broadinstitute.org/ccle/) (40). For the TCGA cohort, gene expression data and associated subtype information were obtained using R package TCGAbiolinks (41, 42). Gene expression levels were measured using log_2_ transformed fragments per kilobase of transcripts per million mapped reads (FPKM). A total of 1079 breast cancer primary tumor samples were included. According to the PAM50 classifier, the number of samples per subtypes were: 1) luminal A (n=506); 2) luminal B (n=207); 3) basal-like (n=190); 4) HER2-enriched (n=82); and 5) normal-like (n=40). PAM50 subtype classification and median centered mRNA expression data of primary tumors from 364 breast cancer patients included in the OSLO2 study were used for expression analyses for the OSLO2 cohort. The OSLO2 study is approved by Regional committees for medical and health research ethics of Norway (approval number 2016/433). The significant differences in gene expression between the five molecular subtypes of breast cancer were examined in both cohorts using One-way ANOVA in GraphPad Prism 9.

Survival analysis was performed using GraphPad Prism 9 using the extracted TCGA cohort data. Patients were divided into a high and low group based on the median expression of *SRGN*.

Correlations between *SRGN* and other genes were analyzed by Pearson correlation in R programing environment (R 4.0.5). The p value of 0.05 (p <0.05), and absolute value of the correlation coefficient above 0.6 (abs (correlation) >0.6) were used for selecting correlated genes with *SRGN*. Gene Set Enrichment Analysis (GSEA) was performed using the web interface (https://www.gsea-msigdb.org/gsea/index.jsp) (43, 44).

Copy number profiling data was obtained from the UCSC Xena platform (https://xenabrowser.net/datapages/). Copy number was measured experimentally using whole genome microarray at a TCGA genome characterization center. Subsequently, the GISTIC2 method was applied using the TCGA FIREHOSE pipeline to produce gene-level copy number estimates. GISTIC2 further thresholded the estimated values to -2, -1, 0, 1, 2, representing homozygous deletion, single copy deletion, diploid normal copy, low-level copy number amplification, or high-level copy number amplification, respectively. Genes were mapped onto the human genome coordinates using UCSC xena HUGO probeMap (45). The estimated values -2 and -1 are classified into loss. The estimated value 1 and 2 are classified into gain. A total of 1042 breast cancer copy number profiling were included in this study. According to the PAM50 classifier, the number of samples per subtypes were: luminal A (n=541); 2) luminal B (n=201); 3) basal-like (n=182); 4) HER2-enriched (n=80); and 5) normal-like (n=38).

### Single Cell RNA-Seq Data Analysis

The publicly available dataset E-MTAB-8107 was used for single cell analysis, which includes 18 breast cancer patients (46). Count matrix of single cell RNA-Seq was analyzed using the Seurat package in R (v4.0.2) to obtain UMAP. The count matrix was already filtered for dying cells by the authors. It was further normalized and scaled regressing out potential confounding factors such as: number of UMIs, number of gene detected in the cell, and percentage of mitochondrial RNA. After scaling, variably expressed genes were used to construct principal components (PCs) and PCs covering the highest variance in the dataset were selected based on elbow and Jackstraw plots to build the UMAP. Clusters were calculated by the FindClusters function with a resolution chosen at 1.2, and visualized using the UMAP dimensional reduction method. Cell type annotation from the authors was used to visualize the distribution of different cell types on UMAP.

### Clinical Samples and Immunostaining

Two tumor micro array (TMA) slides, including a total of 88 patients from the Clinical and Multi-omic (CAMO) cohort were used for immunostaining. The TMA contained samples of both tumor tissue in the invasive front and in the center of the tumor. Patient clinical data was defined as previously described (47). The use of the tissue samples and data was in accordance with the ethical approval from the Regional committees for medical and health research ethics of Norway (approval numbers 2010/1931 and 2013/2271). FFPE sections were baked in a dry oven at 60°C overnight and deparaffinized and rehydrated in xylene and alcohol. The Envison FLEX + system/Dako Autostainer Link 48 (Agilent Technologies) was used for the immunohistochemistry procedure. Antigen retrieval was performed in a commercial pressure chamber (pt-link, Dako) with a heating of 97°C for 20 minutes in Tris/EDTA buffer pH 9. Slides were then allowed to cool to 65°C and permeabilized in TBST (Tris-buffered saline solution containing Tween 20, pH 7.6, Envision Flex Wash Buffer) for 5 minutes. Peroxidase block was performed for 5 minutes in Envision Flex peroxidase block solution followed by protein blocking in 5% BSA for 10 minutes. After incubation with primary anti-serglycin (generated in the lab of Achilleas Theocharis) 1:10 000 for 30 min in room temperature, slides were washed in TBST and incubated with secondary anti-rabbit antibody and linker for 15 minutes. Following a wash in TBST, slides were incubated with EnVision FLEX/HRP (dextran polymer conjugated with horseradish peroxidase) for 20 minutes and washed for 5 minutes. Immunostaining was developed in DAB chromogen solution for 30 minutes. Finally, after washing in TBST, the slides were immediately counterstained with Shandon instant hematoxylin TID, and mounted using eukitt. The immune staining was documented using the Aperio scanner. Only samples defined as luminal A (ER+, HER2-, and Ki67 ≤30) or triple negative (ER-, PR-, HER-) were evaluated. Due to sample loss or poor-quality samples, 42 patients were included in the final scoring. Serglycin expression was scored from 0-3: 0 - no staining, 1 - low intensity, 2 - medium intensity, 3 – high intensity.

## Results

### Serglycin Is Transcriptionally Upregulated During TGF-β Induced Epithelial-Mesenchymal Transition

**Figure 1:**
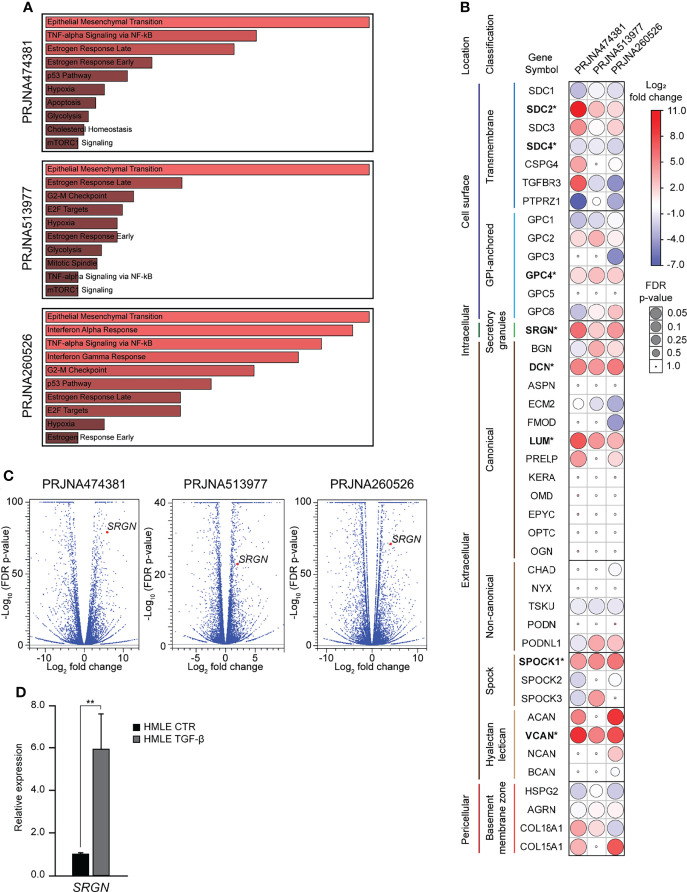
Serglycin is transcriptionally upregulated during TGF-β induced epithelial-mesenchymal transition. **(A–C)** Analysis of RNA-Seq data from three publicly available data sets. **(A)** Gene set enrichment analysis for differentially expressed genes. Length and color represent the significance of that specific gene-set. **(B)** Expression of 42 genes encoding proteoglycans. Bold gene symbols marked with * were defined as differently expressed in all three datasets and with the same direction of change. Color (blue-white-red) symbolizes fold change, while the size of circles symbolizes false discovery rate (FDR) corrected p-value. **(C)** Volcano plot including all analyzed genes. *SRGN* is marked as a red dot in all three plots. **(D)** RT-qPCR data showing *SRGN* expression in TGF- β induced HMLE cells. The experiment includes three biological replicates and is representative for one of a minimum of three independent experimental setups. Error bars represent standard deviation. (**p ≤ 0.01).

Both during normal development and in carcinogenesis, TGF-β is a potent inducer of epithelial-mesenchymal transition (EMT). TGF-β is also important in the regulation of synthesis of many extracellular matrix (ECM) proteins, including several proteoglycans (PGs) (48). In wound healing, TGF-β plays a major role in inducing EMT and inflammation and in in the remodeling of the ECM (49). To investigate the role of proteoglycans (PGs) in TGF-β induced EMT, we mined publicly available RNA-Seq experiments, in which epithelial cells had been stimulated with TGF-β. Our inclusion criteria were: 1) Cells have a normal non-cancerous epithelial origin, 2) the sequencing experiment should include a minimum of two replicates per sample, and 3) the data is published with a complete method description. Three such datasets were identified; PRJNA474381 (37), PRJNA513977 (38), and PRJNA260526 (39). Differential gene expression analysis was performed using the CLC Genomic workbench, and for all three datasets, Gene set enrichment analysis identified EMT as the most significantly enriched hallmark (**Figure 1A**). The data was used to examine the expression of 42 PGs, and we identified eight as significantly differently expressed (**Figure 1B**). Many of the significantly differently expressed PGs have previously been associated with EMT: *SDC2* (8, 9), *SDC4* (10, 11), *SRGN* (12–14), *VCAN* (15–17), *DCN* (18), *LUM* (19), *SPOCK1* (20–23). One of the most significantly differently expressed genes was serglycin (*SRGN*), with a fold change of 59.7 (p=8.8E-80), 4.1 (p=1.6E-23), and 17.7 (p=7.0E-72) (**Figure 1C**). As TGF-β has a functional role in controlling the inflammatory responses (50) and serglycin is an important inflammatory PG, we decided to focus on serglycin in our further studies. To validate the observed induction of serglycin by TGF-β, we treated the HMLE cell line with TGF-β for six days. HMLE is a normal breast epithelial cell line which is known to undergo EMT when stimulated by TGF-β (51, 52). RT-qPCR analysis of *SRGN* showed a 5.9-fold induction (p=0.004) in the HMLE cells after TGF-β stimulation (**Figure 1D**).

### Knockout of Serglycin Causes a Delay in EMT-Associated Gene Expression and Morphology

**Figure 2:**
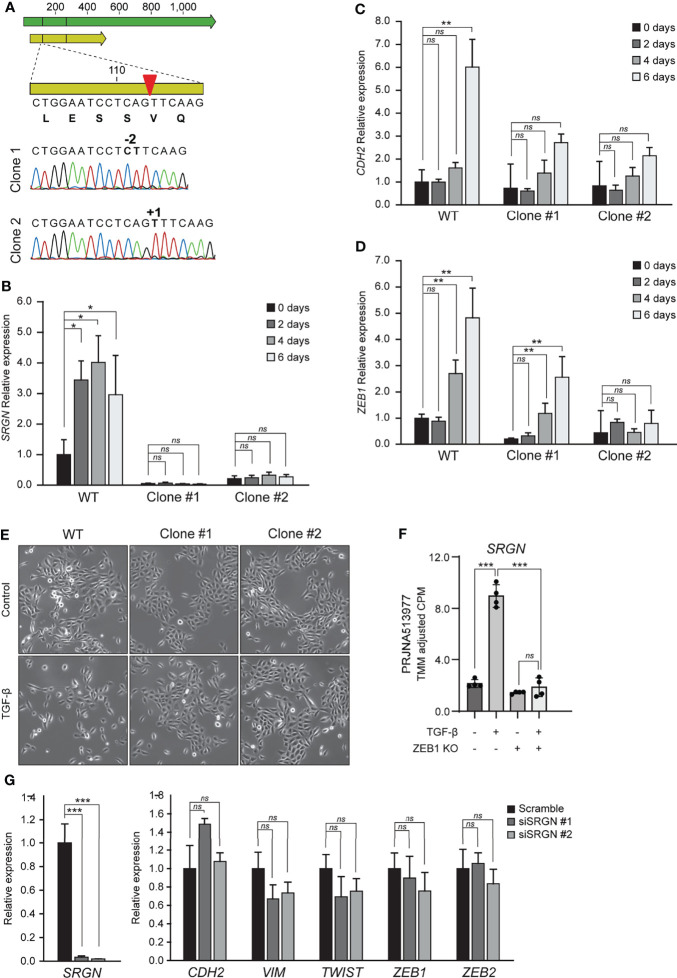
Knockout of serglycin causes a delay in EMT-associated gene expression and morphology. **(A)** Confirmation of CRISPR/Cas9 generation of serglycin knockout clones by Sanger sequencing (bottom part of figure). Green arrow, mRNA; yellow arrow, open reading frame; red arrow, CRISPR/Cas9 target site. **(B–D)** HMLE epithelial cells were stimulated with 10 ng/ml TGF-β for 2, 4, and 6 days and **(B)** *SRGN*, **(C)** *CDH2*, and **(D)** *ZEB1* expression were evaluated by RT-qPCR. **(E)** HMLE epithelial cells were stimulated with 10 ng/ml TGF-β 4 days before imaged using a brightfield microscope. **(F)** Expression of *SRGN* in MCF10A wild type and ZEB1 knockout cells, with and without TGF-β stimulation. **(G)** HMLE mesenchymal cells were transfected for 72 hours with control (scramble) or two siRNAs, siSRGN #1, or siSRGN #2, specifically targeting *SRGN*. *SRGN* expression and expression of mesenchymal marker genes or EMT transcription factors were evaluated by RT-qPCR. For **(B–D)**, and **(G)** the experiments include three biological replicates and are representative for one of a minimum of three independent experimental setups. Error bars represent standard deviation. (***p ≤ 0.001; **p ≤ 0.01; *p ≤ 0.05; ns, not significant).

To examine the functional role of serglycin in TGF-β induced EMT, we generated two HMLE knockout clones by CRISPR/Cas9. The two clones had unique frameshift mutations confirmed by Sanger sequencing (**Figure 2A**). As expected, the serglycin knockout clones had a significantly reduced baseline expression of *SRGN* (**Figure 2B**), as mRNA stability is often reduced by frameshift mutations (53). Further, when treating with TGF-β, the serglycin knockout cells showed no evident induction of *SRGN*, as compared to the wild type HMLE cell line (**Figure 2B**). Importantly, the loss of *SRGN* was accompanied by a reduced induction of N-cadherin (*CDH2*) and the EMT transcription factor *ZEB1* (**Figures 2C, D**). Induction of *SRGN* expression in the normal HMLE cells preceded induction of both *CDH2* and *ZEB1*, and all together these results indicate that upregulation of *SRGN* might be an important early event for efficient EMT induction in HMLE cells. Further, we observed more cells with an elongated mesenchymal phenotype and the growth pattern of the cells were more scattered with less contact with other cells in the wild type control cells as compared to the serglycin knock out cells after TGF-β treatment (**Figure 2E**). As *ZEB1* expression was reduced in the serglycin knockout clones, we examined the relationship between ZEB1 and serglycin in TGF-β induced EMT. One of the previously examined publicly RNA-Seq datasets (PRJNA513977) had included sequencing of an epithelial ZEB1 knockout cell line treated with TGF-β. Knockout of ZEB1 was sufficient to inhibit induction of *SRGN* (**Figure 2F**), indicating that ZEB1 might be a positive regulator of serglycin. Finally, to examine whether serglycin plays a key role in maintaining an EMT phenotype, we transiently transfected mesenchymal HMLE cells with two different *SRGN*-targeting siRNAs. Although the siRNAs significantly silenced the *SRGN* expression, the expressions of key EMT marker genes including *CDH2*, *VIM*, *TWIST*, *ZEB1*, and *ZEB2* remained unchanged 72 hours post-transfection (**Figure 2G**). This suggests that loss of serglycin is not sufficient to revert an established mesenchymal phenotype in the HMLE cell model.

### Breast Cancer Cell Lines With a Mesenchymal Phenotype and the Basal-Like Subtype of Breast Cancer Express High Levels of Serglycin

**Figure 3:**
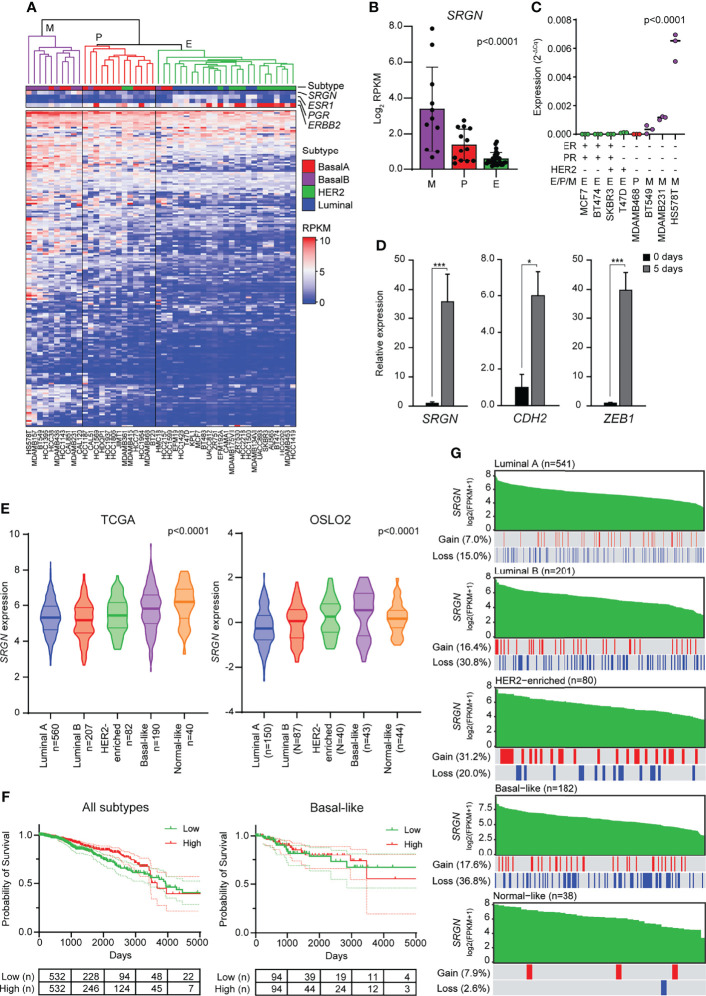
Breast cancer cell lines with a mesenchymal phenotype and the basal-like subtype of breast cancer express high levels of serglycin. **(A)** Hierarchical clustering of 48 breast cancer cell lines based on 194 genes included in the EMT hallmark gene dataset. Subtype and expression of *SRGN*, *ESR1*, *PGR*, and *ERBB2* are shown separately above the heatmap. E, epithelial; P, partial EMT; M, mesenchymal; RPKM, reads per kilobase per million. **(B)** *SRGN* expression in cell lines defined as epithelial **(E)**, partial EMT (P), or mesenchymal (M) based on hierarchical clustering. **(C)** *SRGN* expression in breast cancer cell lines evaluated by RT-qPCR. E, epithelial; P, partial EMT; M, mesenchymal. **(D)** SK-BR-3 cells were stimulated with 20 ng/ml EGF for 5 days. *SRGN*, *CDH2*, and *ZEB1* expression were evaluated by RT-qPCR. The experiment includes three biological replicates and is representative for one of a minimum of three independent experimental setups. Error bars represent standard deviation.**(E)** *SRGN* expression in the TCGA and OSLO2 cohorts. **(F)** Probability of survival for either all breast cancer patients or only patient with the basal-like subtype from the TCGA cohort. High and low expression is based on the median expression value of *SRGN*. Dotted lines represent the 95% confidence interval **(G)** Serglycin copy number gain or loss for patients included in the TCGA cohort. Green bars represent *SRGN* expression, arranged from high to low expression within each subtype. A red line indicates a copy number gain while a blue line represents a copy number loss in the patient with the specific expression value bar seen above in green. FPKM, fragments per kilobase per million. (***p ≤ 0.001; *p, ≤ 0.05).

Elevated levels of TGF-β, both in the plasma and at the invasive front, have been associated with metastasis in breast cancer patients (54–56), and TGF-β has been proposed as a target for cancer therapy (57, 58). Furthermore, many transcription factors and pathways that regulate EMT have been found to be activated in breast tumors (59). To evaluate the association of serglycin expression with EMT in breast cancer, we started by examining RNA-Seq data from 48 breast cancer cell lines deposited to the Cancer Cell Line Encyclopedia (40). The breast cancer cell lines were clustered based on the expression of 194 genes included in the EMT hallmark gene dataset according to the Molecular Signatures Database v7.4 (43, 60) (**Figure 3A**). The clustering gave three main clusters: Mesenchymal (M), partial EMT (P), and epithelial (E) (**Figure 3A**). By examining serglycin mRNA levels, we found that *SRGN* is highest expressed in cell lines defined as mesenchymal, while no or very low expression is identified in epithelial like cells (**Figures 3A, B**). We confirmed by RT-qPCR the higher expression in the mesenchymal breast cancer cell lines Hs578T, MDA-MB-231, and BT549, versus the plastic MDA-MB-468 and the epithelial T-47D, SK-BR-3, BT474, and MCF7 breast cancer cell lines (**Figure 3C**). SK-BR-3, an epithelial like breast cancer cell line, can be induced to go through EMT by EGF stimulation, and show a low baseline expression of *SRGN*. We therefore examined *SRGN* expression in SK-BR-3 during EMT and found that *SRGN* was induced by EGF treatment together with N-cadherin (*CDH2*) and *ZEB1 *(**Figure 3D**).

As serglycin is important for EMT and expressed by mesenchymal like breast cancer cell lines, we went on to examine the expression of *SRGN* in two breast cancer cohorts, TCGA and OSLO2. In both cohorts, *SRGN* is highest expressed in the basal-like subtype of breast cancer (**Figure 3E**), a subtype associated with triple negative breast tumors and tumors with a mesenchymal phenotype (61, 62). However, the expression of *SRGN* was not associated with survival, neither for all patients nor for patients with the basal-like subtype (**Figure 3F**). To examine whether the higher expression of *SRGN* in the basal-like subtype could be a consequence of gene amplification, we analyzed copy-number of *SRGN*. Perhaps surprisingly, a higher incidence of allele loss was seen for the luminal B and basal-like subtypes (30.8% and 36.8%, respectively) as compared with the other subtypes (**Figure 3G**). Generally, allele loss was not seen to be more abundant in patients with lower expression of *SRGN*, and allele gain was not more prominent amongst patients with high expression of *SRGN* (**Figure 3G**), indicating that copy number variation does not influence *SRGN* expression in breast tumor tissue.

### Serglycin Is Expressed by Non-Neoplastic Cells in Breast Tumor Tissue

**Figure 4:**
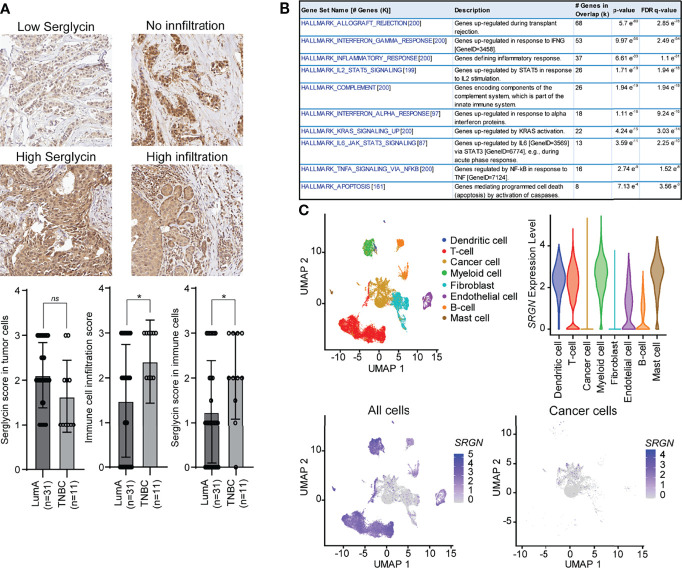
Serglycin is expressed by non-neoplastic cells in breast tumor tissue. **(A)** Immune staining for serglycin in breast cancer patients defined as either luminal A (LumA) or triple negative breast cancer (TNBC). Images representing low (score 1) and high (score 3) serglycin expression in cancer cells, and no (score 0) or high immune cell infiltration (score 3) are included. Bar plots includes quantification of expression of serglycin in either tumor cells or immune cells as well as quantification of immune infiltration in the tumor tissue. Error bars represent semi-standard deviation. **(B)** Gene set enrichment analysis for genes that show a high correlation with *SRGN* expression in patients with the basal-like subtype from the TCGA cohort. **(C)** *SRGN* expression in single cell RNA-Seq data including 18 patients with different subtypes. Top left: Visualization of the different cell types in the UMAP plot. Top right: *SRGN* expression visualized as violine plots per cell type. Bottom left: UMAP plot of *SRGN* expression in all cell types. Bottom right: UMAP plot of *SRGN* expression in only cancer cells. (*p ≤ 0.05; ns, not significant).

The higher *SRGN* genomic loss in the basal-like subtype and the non-enrichment for allele loss in low expressing patients, prompted us to further investigate the association between the basal-like subtype and *SRGN* expression. We therefore performed immune staining on 42 breast cancer patients, either defined as luminal A (ER+, HER2-, and Ki67 ≤30) or triple negative (ER-, PR-, HER-) (**Figure 4A**). In both subtypes, we detected expression of serglycin in both the cancer cells and in infiltrating immune cells. For the cancer cells, a non-significant, but higher expression was observed in the luminal A subtype as compared to the triple negative (**Figure 4A**). In contrast, we observed a significantly higher number of infiltrating immune cells and a significantly higher expression of serglycin in the immune cells in the triple negative subtype as compared to the luminal A subtype (**Figure 4A**). This may indicate that the increased expression of serglycin in the basal-like subtype is a consequence of immune infiltration in the tumor tissue. By performing a co-expression analysis, we found that genes that correlated with *SRGN* expression in the basal-like cases from the TCGA cohort were not enriched for genes associated with EMT, but for genes enriched with the hallmarks Allograft rejection, Inflammatory response, and Complement (**Figure 4B**), which are all associated with an immune response. In addition, the following hallmarks for signaling pathways/molecules were found to be enriched: IFN-γ, STAT5, IFN-α, KRAS, JAK/STAT3, and TNF-α (**Figure 4B**). The observed high expression of serglycin in immune cells, and the association towards an immune response, prompted us to examine *SRGN* expression in a publicly available breast cancer single cell RNA-Seq data set (46). Here, expression analysis showed that *SRGN* is highly expressed in dendritic cells, T-cells, myeloid cells, and mast cells in the tumor tissue, and with lowly detectable expression of *SRGN* in the cancer cells (**Figure 4C**).

## Discussion

**Figure 5:**
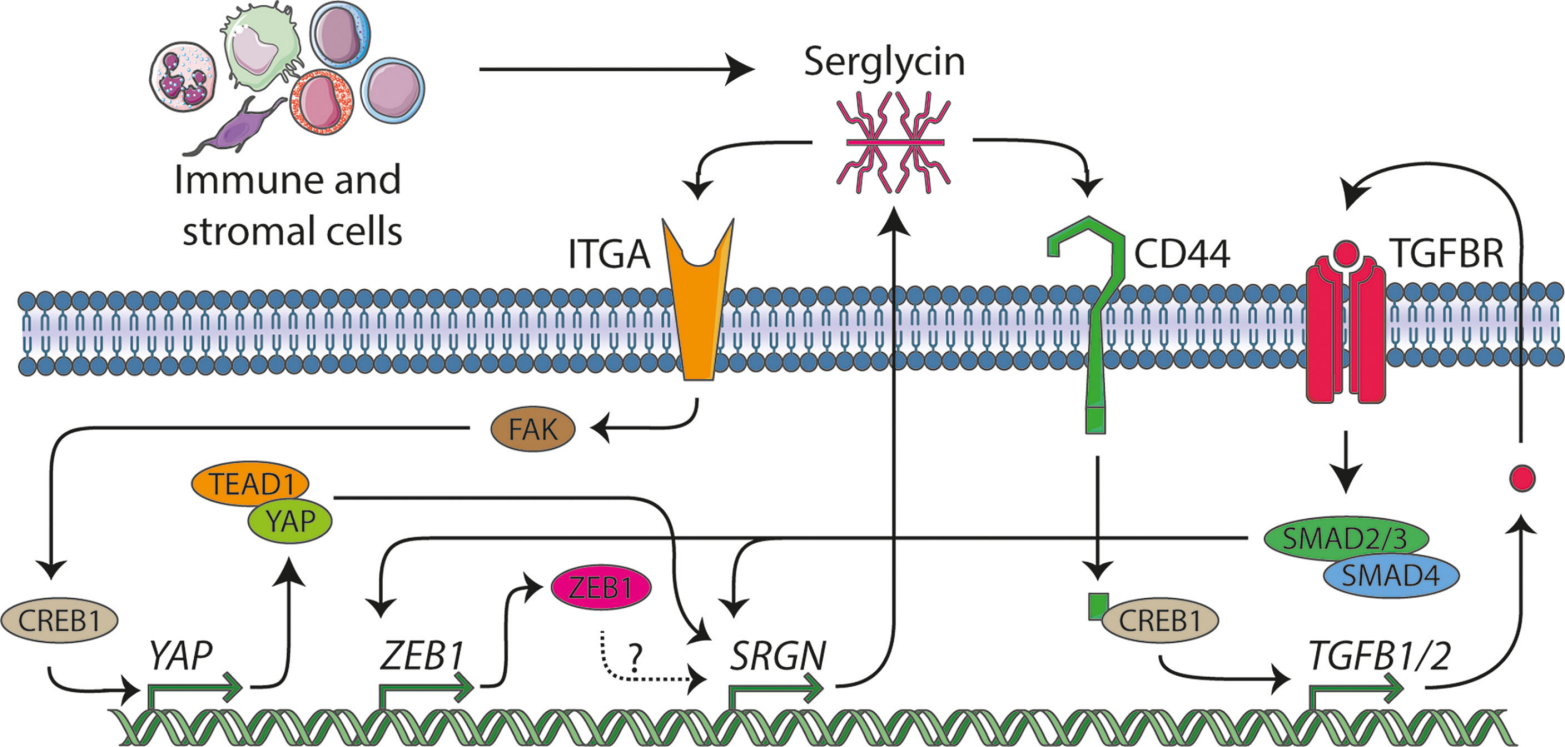
The serglycin signaling network. In this manuscript we identify serglycin to be a TGF-β responsive gene that is induced during epithelial mesenchymal transition (EMT), and we propose that ZEB1 might be a potential transcription factor that can induce serglycin expression. From previous publication, serglycin has been shown to be able to act in an autocrine manner through the TGF-β pathway and *via* the YAP transcriptional co-activator. Several lines of evidence show that immune and stromal cells are major contributors of serglycin in the tumor microenvironment, and we hypothesize that expression of serglycin in the tumor microenvironment can stimulate breast cancer cells to undergo EMT.

EMT in cancer is associated with the acquisition of cancer stem cell-like properties and increased migratory and metastatic abilities, antitumor drug resistance, and immunosuppression. Several stimuli induce EMT in cancer cells including growth factors like TGF-β and EGF, hypoxia, ECM stiffness, cell-cell and cell-ECM interactions, and epigenomic factors (63, 64). We demonstrate that *SRGN* expression is upregulated in different epithelial cells that undergo EMT as response to TGF-β stimulation (**Figure 5**). *SRGN* knockdown in mesenchymal cells is not able to revert an acquired mesenchymal phenotype, but serglycin knockout in epithelial cells exhibit a delay or lower response to TGF-β induced EMT. These data suggest that serglycin is an important player in the EMT reprogramming in epithelial cells, but not essential for cells to maintain a mesenchymal phenotype. The induction of *SRGN* also preceded *CDH2* and *ZEB1*, indicating that *SRGN* induction is an early event in EMT. However, knockout of ZEB1 inhibited *SRGN* induction by TGF-β, suggesting that ZEB1 might be a potentially important transcription factor for serglycin (**Figure 5**). By examining the UCSC Genome Browser, two ZEB1 binding sites are located in the promotor region of serglycin. The potential directly regulation of serglycin by ZEB1 should therefore be experimentally validated. Aggressive breast cancer cells with mesenchymal traits such as MDA-MB-231 and Hs578T, as well as basal-like breast tumors, express high levels of *SRGN*, in contrast to epithelial breast cancer cells and respective luminal subtypes of breast cancer. Altogether, our data point to serglycin as a novel, highly significant marker for breast mesenchymal cells. *SRGN* expression was also found to be induced in the epithelial breast cancer cell line SK-BR-3 through induction of EMT by EGF, which demonstrates the strong association between serglycin and EMT, both in normal as well as in cancer cells.

Previous studies have shown that expression of serglycin in the epithelial breast cancer cell line MCF7 (32) and in non-small cell lung cancer cells (NSCLC) (65) evokes their malignant properties, and that chondroitin sulfate chains attached on the serglycin core protein are required for this regulation. Increased expression of serglycin in epithelial breast cancer cells drives EMT, chemoresistance, proteolytic potential, invasion, and metastasis both *in vitro* and *in vivo* (31, 66–68). Serglycin is constitutively secreted by breast cancer cells acting in an autocrine manner *via* binding to cell surface receptors such as CD44, a key mesenchymal and breast cancer stem cell marker gene, and integrins, possibly through its chondroitin sulfate (CS) chains (69) (**Figure 5**). Binding of serglycin to CD44 activates multiple signaling pathways including CD44/CREB1 to induce TGF-β2 secretion that in turn upregulates *SRGN* expression *via* Smad3 signaling (66), as well as by activating β-catenin signaling (67). Secreted serglycin also triggers ITGA5/FAK/CREB1 signaling to increase the transcription of YAP (68) (**Figure 5**). Reciprocally, YAP/TEAD1 complex promotes the transcription of *SRGN* to form a feed-forward circuit. Furthermore, YAP/RUNX1 complex induces the transcription of HDAC2 to induce chemoresistance and stemness in breast cancer cells (68). Serglycin also promotes IL-8 secretion in breast cancer cells activating IL-8/CXCR2 signaling and underlying signaling cascades such as PI3K, Src, and Rac1 (31). Similarly, to breast cancer, serglycin triggers signaling pathways that evoke malignant properties and EMT in many other cancer types. The serglycin/CD44 signaling axis induces the expression of Nanog and activates NF-κB/claudin-1 axis in NSCLC (70) and triggers MAPK/β-catenin signaling in nasopharyngeal cancer cells (71) to foster EMT, cancer cell stemness, and drug resistance.

Over the last few years, an increasing number of studies have shown that upregulation of serglycin in many tumors is associated with aggressive tumor cells properties (6). In breast cancer, serglycin has been demonstrated to be associated with chemotherapy resistance (31, 72). Here, we describe that *SRGN* expression in the basal-like subtype of breast cancer is correlated to the expression of genes involved in inflammatory response. Previous reports have also highlighted the role of serglycin in regulation of the biosynthesis of inflammatory mediators and growth factors in immune cells, platelets, endothelial, and tumor cells (2, 69). Serglycin also controls the proteolytic potential of tumor cells *via* regulating the expression and activity of MMPs (6, 12, 31, 73). Both inflammatory mediators and MMPs are known to be involved in EMT and in the advancement of the malignant phenotype of breast cancer cells (74, 75). We have demonstrated that the high expression of *SRGN* in the generally aggressive basal-like breast cancer subtype is not a consequence of gene amplification, but rather surprisingly, the subtype shows a higher probability for allele loss. This may imply that the bulk of serglycin expression comes from stromal cells (**Figure 5**). Several cell types populate the tumor stroma, with the most predominant being immune cells, cancer-associated fibroblasts, and endothelial cells. Breast cancer single cell RNA-Seq data indicate that *SRGN* is highly expressed mostly in stromal cells and lymphocytes, including myeloid, dendritic, mast cells, T-lymphocytes, endothelial cells, and B-lymphocytes. Cancer cells and fibroblasts exhibit low *SRGN* expression in the analyzed sample cohort. This points toward infiltrating immune cells being the major source of serglycin in breast tumor tissue. It is well known that serglycin is constitutively expressed by immune cells such as macrophages, lymphocytes, and mast cells and its expression is upregulated upon their activation (2, 76). Mast cells infiltrate the glioma microenvironment and secrete high levels of serglycin, and the process is associated with poor survival (77). Co-culture of mast cells with glioma cells induces the expression of serglycin, CD44, ZEB1, vimentin, CXCL10, CXCL12, and TNF-α in glioma cells and IL-6 and CXCL1 in mast cells, creating an inflammatory milieu that induce glioma cell aggressiveness (77). In glioblastoma, serglycin works in an autocrine manner, and suppression of serglycin potently reduces their malignant properties, pro-neoplastic signaling, and stemness, and evokes astrocytic differentiation (12). This demonstrates a key role for serglycin in glioma progression, and demonstrates how serglycin, either *via* induction by an inflammatory response or by direct secretion by immune cells is of major importance for cancer progression. Serglycin is also upregulated in cancer-associated fibroblasts in hypoxic conditions and its secretion activates Wnt/β-catenin signaling, leading to enhanced cancer cell stemness, chemoresistance, and tumor growth (78).

It is proposed that cancer cells that are subjected to partial EMT and remain in a hybrid state where they co-express both epithelial and mesenchymal markers, have the greatest malignant and metastatic potential (63, 79). Furthermore, subpopulations of cancer cells composed of epithelial, hybrid, or mesenchymal cells have been found to be localized in specific microenvironments where distinct stromal cells reside (79). Tumor areas specifically populated by the hybrid or mesenchymal cancer cells have an increased number of immune cells, particularly monocytes and macrophages, lymphocytes, and endothelial cells, highlighting the association of EMT induction with the inflammatory environment. Interestingly, cancer cells express increased levels of chemokines and other pro-inflammatory and pro-angiogenic molecules that attract stromal cells and coordinates their niche formation and spatial organization (79). Further, serglycin has recently been identified to be involved in inflammatory reactions in adipose tissue (80). Altogether, our data and previously published data on serglycin, EMT, and cancer, may imply that serglycin participates in an immune cell-cancer cell crosstalk, acting as a regulatory molecule that links inflammation and induced oncogenic signaling and EMT in breast cancer cell (**Figure 5**). Future studies should therefore investigate the molecular mechanisms behind this potential crosstalk, *via* serglycin, between the breast cancer cells and the infiltrating immune cells and if expression of serglycin by the immune cells can induce EMT in the breast cancer cells.

## Data Availability Statement

The original contributions presented in the study are included in the article/supplementary material. Further inquiries can be directed to the corresponding author.

## Ethics Statement

The studies involving human participants were reviewed and approved by Regional committees for medical and health research ethics of Norway. The patients/participants provided their written informed consent to participate in this study.

## Author Contributions

MT-G, TR, BH, SN, and EK conducted experiments. XT, MC, LN, and EK collected and analyzed data. LM, L-TB, GM, MP, AT, SK, and EK provided material or patient samples. MP, AT, SK, and EK wrote the manuscript. All authors revised and approved the manuscript.

## Funding

EK is funded by Helse Nord (HNF1585-21), SK is funded by the Throne Holst Foundation, LN is funded by Helse Sør-Øst (2017034), and GM is funded by the Norwegian Cancer Society (#190257).

## Conflict of Interest

The authors declare that the research was conducted in the absence of any commercial or financial relationships that could be construed as a potential conflict of interest.

The handling editor declared a past co-authorship with the author ADT.

## Publisher’s Note

All claims expressed in this article are solely those of the authors and do not necessarily represent those of their affiliated organizations, or those of the publisher, the editors and the reviewers. Any product that may be evaluated in this article, or claim that may be made by its manufacturer, is not guaranteed or endorsed by the publisher.
